# Application of Mamdani Fuzzy Inference System in Poultry Weight Estimation

**DOI:** 10.3390/ani13152471

**Published:** 2023-07-31

**Authors:** Erdem Küçüktopçu, Bilal Cemek, Halis Simsek

**Affiliations:** 1 Department of Agricultural Structures and Irrigation, Ondokuz Mayıs University, Samsun 55139, Türkiye; bcemek@omu.edu.tr; 2 Department of Agricultural and Biological Engineering, Purdue University, West Lafayette, IN 47907, USA; simsek@purdue.edu

**Keywords:** artificial intelligence, broiler, defuzzification, expert system, linguistic variables

## Abstract

**Simple Summary:**

With the rapid technological advances, the application of artificial intelligence (AI) has witnessed significant growth in the agricultural industry, specifically in the poultry sector. The use of AI in estimating poultry weight can significantly impact production economics and overall efficiency in the poultry sector. Therefore, this paper presents an innovative AI approach based on the fuzzy logic (FL) method for estimating poultry weight. The FL models were created using expert knowledge and key input variables such as indoor temperature, humidity, and feed consumption. This study’s findings demonstrate that FL-based methods exhibit great promise for achieving accurate and efficient poultry weight estimation. Integrating the FL technique in the poultry industry can bring numerous benefits, including improved decision-making processes, enhanced efficiency, and reduced costs.

**Abstract:**

Traditional manual weighing systems for birds on poultry farms are time-consuming and may compromise animal welfare. Although automatic weighing systems have been introduced as an alternative, they face limitations in accurately estimating the weight of heavy birds. Therefore, exploring alternative methods that offer improved efficiency and precision is necessary. One promising solution lies in the application of AI, which has the potential to revolutionize various aspects of poultry production and management, making it an indispensable tool for the modern poultry industry. This study aimed to develop an AI approach based on the FL model as a viable solution for estimating poultry weight. By incorporating expert knowledge and considering key input variables such as indoor temperature, indoor humidity, and feed consumption, FL-based models were developed with different configurations using Mamdani inferences and evaluated across eight different rearing periods in Samsun, Türkiye. This study’s results demonstrated the effectiveness of FL-based models in estimating poultry weight. The models achieved varying average absolute error values across different age groups of broilers, ranging from 0.02% to 5.81%. These findings suggest that FL-based methods hold promise for accurate and efficient poultry weight estimation. This study opens up avenues for further research in the field, encouraging the exploration of FL-based approaches for improved poultry weight estimation in poultry farming operations.

## 1. Introduction

The increasing world population is expected to pose significant challenges for meeting the growing food demand in the coming years [1,2]. Poultry meat production has proven to be a crucial factor in meeting this demand due to its ability to efficiently produce high-quality protein, making it a key player in the global food system [3]. The world’s chicken meat production has been continuously expanding since 2015 and reached 102 million tons in 2022 due to its affordable prices and superior quality nutrition [4]. Although projected to rise more slowly in the future than in the past decade, poultry meat is expected to continue to be the primary growth driver for meat production [5].

The physical growth attributes of poultry, including their size and body weight, are crucial factors in evaluating the efficiency of poultry production [6]. This evaluation involves comparing the amount of feed consumed with the measured weight of the birds. It is also essential for flock managers to know the average weight and weight range at slaughter to select and slaughter broilers accurately. Accurate weight estimation of poultry is crucial for efficiently managing poultry farming operations. Poultry farming is essential for the livestock industry, providing a significant portion of the world’s meat products. With the rapid expansion of poultry enterprises to meet the growing demand for meat products, the importance of monitoring the weight of poultry has increased significantly [7].

The most common method of measuring weight is a manual process that is labor-intensive, compromises chicken welfare, and can lead to lower quality and yield and even death of the birds [8]. Automatic weighing systems in modern poultry houses weigh birds where they volunteer, but heavier birds are less likely to visit the platform, making it difficult to determine their weight accurately [9]. To overcome such challenges, researchers have developed weight estimation models [10,11] but rely on empirical formulas based on linear regressions that may not fully capture the complex and nonlinear relations among factors that influence bird weight [12]. Therefore, alternative approaches are needed to improve the accuracy and reliability of weight estimation in poultry.

FL offers a promising alternative approach to poultry weight estimation, as it is capable of handling uncertain and imprecise data while capturing complex, nonlinear relations among variables. The methodology of FL involves utilizing a set of linguistic rules to generate an output by combining various input variables [13]. The primary objective of this approach is to establish a common linguistic platform that links a set of words, representing qualitative assessments provided by an expert, to a numerical set, representing quantitative data. This process aims to bridge the gap between qualitative and quantitative information, thereby facilitating a more comprehensive analysis and informed decision making [14].

By integrating expert knowledge and experience, FL systems enhance the accuracy of weight estimation, even when dealing with incomplete or uncertain data. This ability to account for uncertainty and vagueness in the data makes FL a valuable tool for poultry weight estimation, allowing for more reliable results in practical applications [15].

Accurate weight estimation is critical to optimizing production efficiency, minimizing waste, and ensuring profitability. The use of FL poultry weight estimation systems can help meet the increasing demand for food by improving production capacity and quality in the poultry industry. In this study, an AI model for poultry weight prediction was developed using an FL approach based on this premise.

### Literature Review, Research Questions, and Contributions of the Study

AI techniques for poultry management have gained popularity worldwide due to their ability to accurately model complex relations among various factors [15,16,17,18,19]. These techniques can handle imprecise and uncertain data and adapt to changing conditions, making them a powerful tool in poultry production [19,20,21].

The poultry industry is an important sector in Türkiye, and the demand for poultry products is rapidly increasing [22]. However, AI techniques for poultry weight estimation are not yet widely used in Türkiye. By adopting AI techniques, including FL expert systems, for poultry weight estimation, the Turkish poultry industry can remain competitive and sustainable in the long term, especially as the demand for poultry products continues to increase worldwide.

The systematic application of FL is essential for solving complicated real-life problems, especially for decision making. From a human perspective, tools are needed to manage subjective information, while from a data processing perspective, precise definitions are required to facilitate decision making. However, humans can work with vague, imprecise, and linguistic definitions commonly observed in the real world. Therefore, the inclusion of FL enables handling such complex, uncertain, and imprecise information and can improve the accuracy and reliability of decision-making processes [15,23,24,25].

The literature presents numerous studies that utilize various AI techniques for modeling poultry production data. For example, the authors of [26] implemented a three-layer feedforward artificial neural network to predict body weight in broiler chickens. In ref. [27], the author employed neural network models to predict the growth of broiler chickens over the next 50 days using actual growth data. In the research of [28], a novel approach was proposed for modeling the growth of broiler chickens by introducing a differential recurrent neural network. The researchers in [29] explored the feasibility of analyzing the growth of chickens using a radial basis function neural network approach. In ref. [30], the author developed an artificial neural network model that effectively established the relations between the input and output variables of feed intake, weight gain, and feed conversion ratio. In the investigation of [31], dynamic neural network forecasting models were trained on farm-scale broiler batch production data from 12 batches within the same house. These models were used to forecast future broiler weight while incorporating environmental conditions such as heating, ventilation, temperature, and behavioral factors like feed and water consumption. The study’s findings indicated that the model successfully captured the dynamic interconnection between environmental conditions and broiler growth, highlighting the model’s ability to accurately predict the future growth patterns of broilers based on the provided inputs. The authors of [32] demonstrated that their neural network model achieved satisfactory performance in predicting the feed conversion of broilers under heat stress.

These studies have contributed significantly to our understanding; however, to the best of our knowledge, this study is a pioneering study that, for the first time, reports the use of FL systems for estimating broiler weights in poultry buildings. Particularly, this study addresses the following research questions: (1) What is the predictive ability of FL systems in estimating bird weights in poultry houses? (2) What is the effect of the different defuzzification methods in the developed FL model for weight estimation? (3) What potential impact will the developed FL model have on the poultry industry? Various research methods were used to address these research questions. After conducting the literature survey and finding the research gaps, the main contribution of the current work will be to provide a novel method for estimating the broiler weights in poultry buildings based on an FL approach.

The remaining sections of the article are organized as follows: Section 2 describes the methodology proposed in the study, detailing the approach for estimating broiler weights in poultry buildings. Section 3 presents the results obtained from implementing the proposed methodology. It includes the findings related to broiler weight estimation using the FL systems and any additional analyses. The discussion section (Section 4) analyzes and interprets the results obtained. It provides insights into the implications of the findings, explores potential limitations faced during the study, and compares the outcomes with previous research. Section 5 summarizes the main findings of the study and highlights their significance. Additionally, it offers recommendations for future research directions.

## 2. Materials and Methods

### 2.1. Experimental Building and Data

The research was conducted at a commercial broiler farm located in Samsun, Türkiye (41°70′ N, 36°30′ E). The farm had the dimensions of 90 m in length, 14 m in width, and 3.80 m in height. Chickens from the “Ross 308” breeding stock were reared until they reached 40–42 days of age. The farm utilized an automatic control system to regulate ventilation, heating, lighting, feeding, and watering. Chicken weights and feed consumption were recorded weekly over eight rearing seasons. The statistical parameters of indoor temperature, indoor relative humidity, feed consumption, and chicken weights are shown in Table 1.

### 2.2. Fuzzy Logic (FL)

FL was introduced by ref. [33] in the 1960s as a mathematical framework for dealing with uncertainty and ambiguity in decision making. FL extends classical (crisp) set theory by allowing degrees of membership or truth values between 0 and 1 rather than just binary membership (either 0 or 1), as in classical set theory [33].

FL is an expert system composed of fuzzification, knowledge base, inference engine, and defuzzifier parts [34,35]:

Fuzzifier: It converts crisp input values (e.g., numerical values) into fuzzy values, representing degrees of membership in fuzzy sets. The fuzzifier takes input values and maps them to fuzzy sets using membership functions, which define the shape of the fuzzy sets and determine how input values are transformed into fuzzy values.

Knowledge Base: It contains the fuzzy rules and the associated fuzzy sets. Fuzzy rules are typically expressed in the form of “If–Then” statements, where the antecedent (input) part consists of fuzzy sets and the consequent (output) part consists of fuzzy sets or crisp values. The knowledge base stores these rules, which are used for making decisions or performing actions based on the fuzzy input values.

Inference Engine: It is responsible for applying the fuzzy rules to the fuzzy input values to determine the fuzzy output values. The inference engine uses FL operations, such as AND, OR, and NOT, to combine the fuzzy sets and perform fuzzy reasoning. The result is a set of fuzzy output values representing the degrees of membership in fuzzy sets for the output variables.

Defuzzifier: It is responsible for converting the fuzzy output values into crisp output values. The defuzzifier takes the fuzzy output values and maps them back to crisp values using various methods, such as centroid, mean of maximum, or weighted average. The crisp output values represent the final decisions or actions based on the fuzzy input values and the fuzzy rules.

### 2.3. Fuzzification

Membership functions are determined as the initial step in designing a Mamdani fuzzy inference system (MFIS), considering the fact that fuzzy inputs are used [36]. The membership functions can take different functional forms to represent different degrees of fuzziness, such as linear, concave, and exponential forms. Among these, the linear triangular and linear trapezoidal membership functions are commonly used in many studies [37].

**Figure 1:**
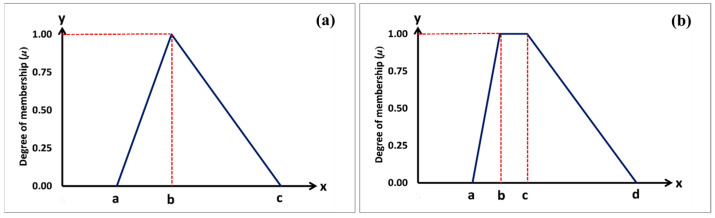
(**a**) Triangular and (**b**) trapezoidal membership functions.

A triangular fuzzy number is defined by a membership function that assigns membership degrees to values within a certain range (Figure 1a). The membership function for a triangular fuzzy number with the triplet (*a*, *b*, *c*) can be described as follows:

(1) $$\mu_{A}\left(x\right)=\left\{\begin{array}{l} x\leq a\;or\;\;x\geq c;\;0\\ a<x<b;\;\left(x-a)/(b-a\right)\\ b<x<c;\;\left(c-x)/(c-b\right)\\ x=b;\;1 \end{array}\right.$$

A trapezoidal fuzzy number is characterized by a membership function that assigns membership degrees to values within a specific range (Figure 1b). The membership function for a trapezoidal fuzzy number with the vertices (*a*, *b*, *c*, *d*) can be defined as follows:

(2) $$\mu_{A}\left(x\right)=\left\{\begin{array}{l} x\leq a\;\;or\;x\geq d;\;0\\ a<x<b;\;\left(x-a)/(b-a\right)\\ c<x<d;\;\left(d-x)/(d-c\right)\\ b\leq x\leq c;\;1 \end{array}\right.$$

### 2.4. Fuzzy Rule Base

The establishment of rules holds a central position within a fuzzy inference system (FIS). These rules are typically expressed using linguistic terms, offering a more intuitive representation compared to numerical terms. The commonly adopted format for these rules is the “If–Then” structure, which applies well to implementing fuzzy conditional statements. The utilization of ‘‘If–Then’’ rules is crucial because they provide a means to effectively capture and embody human expertise and knowledge within a fuzzy framework [38]. When it comes to deriving fuzzy rules, various approaches exist, including integrating expert knowledge and applying fuzzy modeling techniques. The present study implemented a fuzzy model by defining a set of fuzzy linguistic rules based on expert knowledge.

### 2.5. Inference Engine and Defuzzification

The fuzzy interface engine is an essential component of an FL system that processes the inputs and applies the rules to produce a meaningful output. It combines the linguistic variables and fuzzy sets identified in the previous steps to make a decision.

The integration process combines the individual fuzzy areas determined in the previous step to generate the overall fuzzy conclusion. This process involves aggregating the fuzzy sets, considering the weights assigned to each rule and output fuzzy set.

The fuzzy inference process used in this step typically employs the “min–max inference” method. This method uses the minimum and maximum operators to determine the degree of membership of the output variable in the fuzzy sets. The minimum operator is used to find the degree of membership of the input variable in the corresponding fuzzy set, while the maximum operator is used to determine the degree of membership of the output variable in the fuzzy set associated with the rule [39].

The result of the integration step is a fuzzy conclusion that provides an output value or a range of values. This output value is then defuzzified to obtain a crisp value that can be used as the final decision or as an input to another system. There are several popular defuzzification approaches, including the center of area method (COA), the bisector of area method (BOA), the mean of maximum method (MOM), the smallest of maximum method (SOM), and the largest of maximum method (LOM) [40]. This paper used these defuzzification methods to prove the model’s validity. The predictive abilities of these methods were investigated according to the coefficient of determination (*R^2^*), mean absolute error (*MAE*), and root mean square error (*RMSE*). The equations are defined below:

(3) $$R^{2}=\frac{\sum_{i=1}^{n}\left(X_{mea}-X_{mea,avg})(X_{est}-X_{est,avg}\right)}{\sum_{i=1}^{n}{\left(X_{mea}-X_{mea,avg}\right)}^{2}\sum_{i=1}^{n}{\left(X_{est}-X_{est,avg}\right)}^{2}}$$

(4) $$MAE=\frac{\sum_{i=1}^{n}\left(\left|X_{mea}-X_{est}\right|\right)}{n}$$

(5) $$RMSE=\sqrt{\frac{\sum_{i=1}^{n}{\left(X_{mea}-X_{est}\right)}^{2}}{n}}$$

In addition, the prediction of the MFIS model was made according to the absolute percentage error (*APE*) as follows:

(6) $$APE=\frac{\left|X_{mea}-X_{est}\right|}{X_{mea}}\times 100$$

where *X_mea_* is the measured value of variables, *X_est_* is the estimated value of variables, and *X_mea,avg_* and *X_est,avg_* are the measured and estimated average values of variables, respectively. *n* is the sample number.

## 3. Results

**Figure 2:**
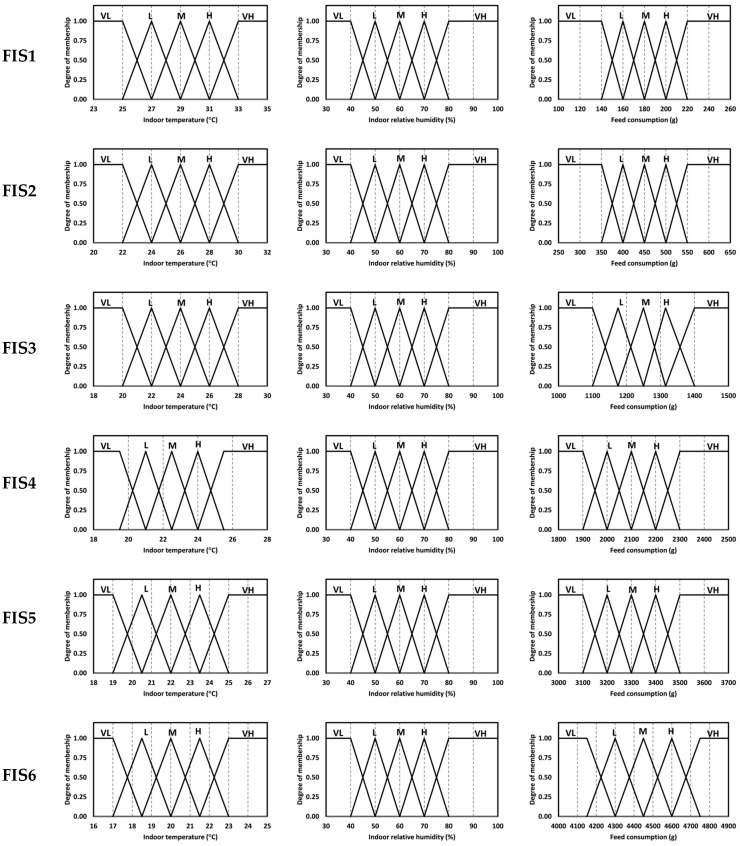
Membership functions for inputs.

**Figure 3:**
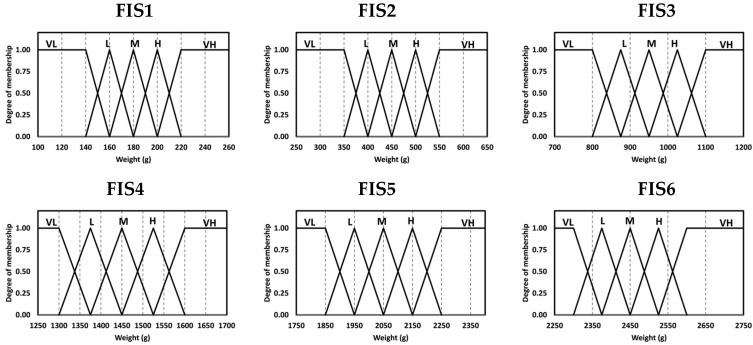
Membership functions for output.

The determination of membership functions is a critical step in developing an accurate and reliable FIS. It requires expert knowledge, careful analysis, and sound judgment. This paper used triangular and trapezoidal membership functions due to their simplicity and good computational efficiency. We employed five fuzzy sets of membership functions for the inputs and outputs of the MFIS (FIS 1, FIS 2, FIS 3, FIS 4, FIS 5, and FIS 6). The fuzzy sets were represented in the form of linguistic rating variables, such as ‘‘Very Low’’, ‘‘Low’’, ‘‘Medium’’, ‘‘High’’, and ‘‘Very High’’ for the weeks of the rearing period, as shown in Table 2. The memberships of the inputs and output of the MFIS are illustrated in Figure 2 and Figure 3, respectively.

These membership functions helped in converting numeric variables into linguistic terms. For example, some linguistic expressions and membership functions of FIS1 obtained from the developed rules were given as follows:

For indoor temperature (*ti*): Very Low (VL) and Medium (M) linguistic variables,

(7) $$\begin{array}{l} \mu_{VL}(ti)=\left\{\begin{array}{l} 23\leq ti\leq 25;\;1\\ 25<ti<27;\;(27-ti)/2\\ ti\geq 27;\;0 \end{array}\right.\\ \mu_{VL}(ti)=\left\{\frac{1.00}{25.00}+\frac{0.90}{25.20}+\cdots +\frac{0.50}{26.00}+\cdots +\frac{0.10}{26.80}+\frac{0.00}{27.00}\right\} \end{array}$$

(8) $$\begin{array}{l} \mu_{M}(ti)=\left\{\begin{array}{l} ti\leq 27\;or\;ti\geq 31;\;0\\ 27<ti<29;\;(ti-27)/2\\ 29<ti<31;\;(31-ti)/2\\ ti=29;\;1 \end{array}\right.\\ \mu_{M}(ti)=\left\{\frac{0.00}{27.00}+\frac{0.10}{27.20}+\cdots +\frac{1.00}{29.00}+\cdots +\frac{0.10}{30.80}+\frac{0.00}{31.00}\right\} \end{array}$$

For indoor relative humidity (*rhi*): Low (L) and High (H) linguistic variables,

(9) $$\begin{array}{l} \mu_{L}(rhi)=\left\{\begin{array}{l} rhi\leq 40\;or\;rhi\geq 60;\;0\\ 40<rhi<50;\;(rhi-40)/10\\ 50<rhi<60;\;(60-rhi)/10\\ rhi=50;\;1 \end{array}\right.\\ \mu_{L}(rhi)=\left\{\frac{0.00}{40.00}+\frac{0.10}{41.00}+\cdots +\frac{1.00}{50.00}+\cdots +\frac{0.10}{59.00}+\frac{0.00}{60.00}\right\} \end{array}$$

(10) $$\begin{array}{l} \mu_{H}(rhi)=\left\{\begin{array}{l} rhi\leq 60\;or\;rhi\geq 80;\;0\\ 60<rhi<70;\;(rhi-60)/10\\ 70<rhi<80;\;(80-rhi)/10\\ rhi=70;\;1 \end{array}\right.\\ \mu_{H}(rhi)=\left\{\frac{0.00}{60.00}+\frac{0.10}{61.00}+\cdots +\frac{1.00}{70.00}+\cdots +\frac{0.10}{79.00}+\frac{0.00}{80.00}\right\} \end{array}$$

For feed consumption (*fc*): Medium (L) and Very High (VH) linguistic variables,

(11) $$\begin{array}{l} \mu_{M}(fc)=\left\{\begin{array}{l} fc\leq 160\;or\;fc\geq 200;\;0\\ 160<fc<180;\;(fc-160)/20\\ 180<fc<200;\;(200-fc)/20\\ fc=180;\;1 \end{array}\right.\\ \mu_{M}(fc)=\left\{\frac{0.00}{160.00}+\frac{0.10}{162.00}+\cdots +\frac{1.00}{170.00}+\cdots +\frac{0.10}{198.00}+\frac{0.00}{200.00}\right\} \end{array}$$

(12) $$\begin{array}{l} \mu_{VH}(fc)=\left\{\begin{array}{l} fc\leq 200;\;0\\ 200<fc<220;\;(fc-200)/20\\ 220\leq fc\leq 260;\;1 \end{array}\right.\\ \mu_{VH}(fc)=\left\{\frac{0.00}{200.00}+\frac{0.10}{202.00}+\cdots +\frac{0.50}{210.00}+\cdots +\frac{0.90}{218.00}+\frac{1.00}{220.00}\right\} \end{array}$$

For weight (*wa*): Very Low (VL) and Very High (VH) linguistic variables,

(13) $$\begin{array}{l} \mu_{VL}(wa)=\left\{\begin{array}{l} 100\leq wa\leq 140;\;1\\ 140<wa<160;\;(160-wa)/20\\ wa\geq 160;\;0 \end{array}\right.\\ \mu_{VL}(wa)=\left\{\frac{1.00}{140.00}+\frac{0.90}{142.00}+\cdots +\frac{0.50}{150.00}+\cdots +\frac{0.10}{158.00}+\frac{0.00}{160.00}\right\} \end{array}$$

(14) $$\begin{array}{l} \mu_{VH}(wa)=\left\{\begin{array}{l} wa\leq 200;\;0\\ 200<wa<220;\;(wa-200)/20\\ 220\leq wa\leq 260;\;1 \end{array}\right.\\ \mu_{VH}(wa)=\left\{\frac{0.00}{200.00}+\frac{0.10}{202.00}+\cdots +\frac{0.50}{210.00}+\cdots +\frac{0.90}{218.00}+\frac{1.00}{220.00}\right\} \end{array}$$

After determining the fuzzy sets in the form of linguistic rating variables, the next step was to establish rules based on expert knowledge. In this case, a total of 97 possible rules were formed from the six FISs: 10 rules for FIS1, 20 rules for FIS2, 15 rules for FIS3, 15 rules for FIS4, 17 rules for FIS5, and 20 rules for FIS6. For example, if the indoor temperature is “Low”, indoor relative humidity is “Low”, and feed consumption is also “Very Low”, then the weight for FIS1 output is “Very Low”. Another example of a rule is, if the indoor temperature is “Medium”, indoor relative humidity is “Medium”, and feed consumption is also “Very High”, then the weight for FIS6 output is “High”. However, since there were too many rules to display, only some of the dominant rules in the proposed model are listed in Table 3.

**Figure 4:**
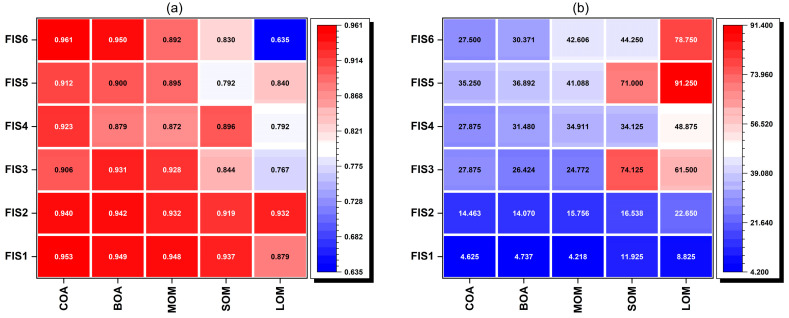

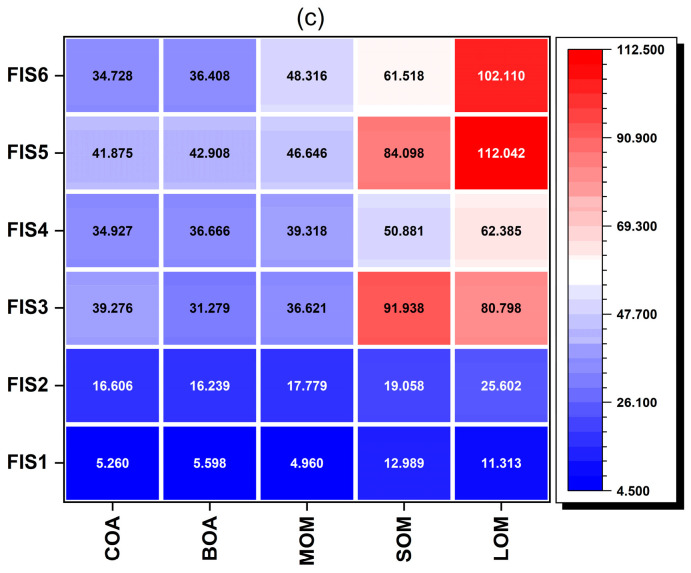
(**a**) R^2^, (**b**) MAE, and (**c**) RMSE values of the different defuzzification methods.

In the last step of the MFIS, a defuzzification process was performed to obtain crisp values. We applied various defuzzification methods, including COA, BOA, MOM, SOM, and LOM, to demonstrate the model’s validity. The R^2^, RMSE, and MAE results of these models are presented in Figure 4a–c. These figures indicate that the COA defuzzification method outperformed the others based on the R^2^, RMSE, and MAE criteria. The BOA, MOM, and SOM methods followed closely behind, while the LOM method was the least accurate model. Therefore, the COA method was used for the defuzzification process in further experiments.

The measured and estimated broiler weight values and their corresponding APE values are presented in Table 4. The APE values for FIS1, FIS2, FIS3, FIS4, FIS5, and FIS6 ranged between 0.02–5.78%, 0.21–5.81%, 0.12–4.84%, 0.41–4.29%, 0.15–3.66%, and 0.37–2.47%, respectively, depending on the season.

**Figure 5:**
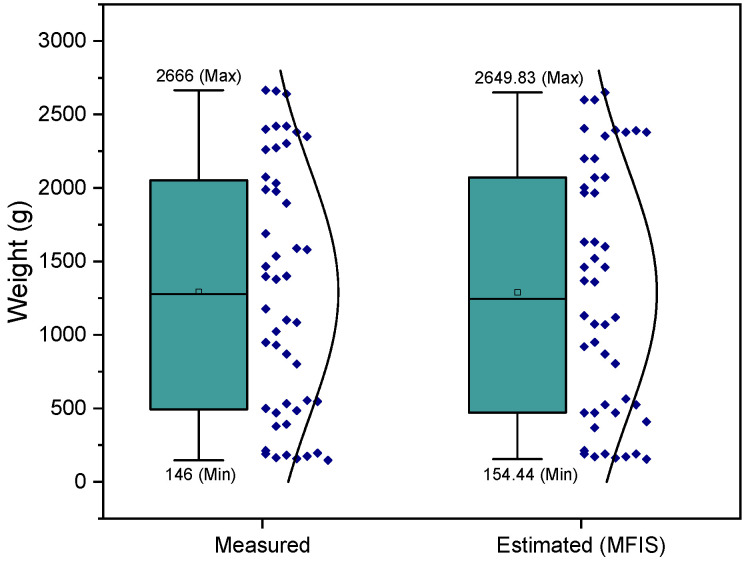
Box plot—measured vs. estimated (MFIS).

Figure 5 illustrates box plots that depict the distribution of the data. As shown in the figure, the measured weight values ranged from 146 g to 2666 g, while the estimated values ranged from 154.4 g to 2649.83 g. The data indicated that the MFIS models exhibited higher accuracy in predicting broiler weight.

## 4. Discussion

Physical growth characteristics are critical factors that determine the efficiency of poultry production. Body weight is a crucial indicator of overall poultry health and development and is a key factor in determining the market value of poultry [6]. Traditional methods of measuring poultry weight can compromise bird welfare and may even lead to lower quality and yield or death [8]. FL represents a promising alternative approach to poultry weight estimation that can handle uncertain and imprecise data while modeling complex, nonlinear relations among variables.

AI models for poultry growth prediction offer several advantages over traditional methods, including faster predictions, greater accessibility, and the ability to simultaneously analyze a wide range of variables [20,41,42]. However, it is crucial to handle the data appropriately to ensure that the model’s predictions are accurate and unbiased.

The application of AI models in poultry growth prediction is expanding. For instance, in the investigation of [17], the utilization of AI models with various training inputs resulted in a prediction accuracy of growth and body weight in broiler chickens ranging from 98% to 99%. In another study, conducted for ref. [26], the AI approach appeared highly satisfactory, and the performance was very high, at approximately 99%. This level of performance was deemed acceptable by broiler researchers. The authors of [43] aimed to enhance the prediction of live weight using various artificial neural network techniques, such as Bayesian regulation, Levenberg–Marquardt, scaled conjugate gradient, and gradient descent. Among these, Bayesian regulation exhibited the best performance with an R^2^ value of 0.98 for predicting broiler weight. Ref. [27] utilized various neural network models to forecast the growth of broiler chickens for a subsequent 50 days, based on actual growth data. The study concluded that differences between the response of the neural network models and the actual poultry could be attributed to a variety of factors, including the pattern of the data, the examples used for training and testing, the architecture of the network, and the training process of each model.

These above-mentioned studies can provide valuable insights into the factors that influence poultry growth and help producers optimize their operations for greater efficiency and profitability. However, no study has been conducted about using FL systems to estimate poultry building broiler weights. This paper introduces a novel FL-based approach for estimating broiler weights in poultry buildings. The proposed method achieved mean absolute percentage errors (MAPEs) of 2.83%, 2.95%, 2.52%, 2.08%, 2.03%, and 1.20% based on rearing weeks, demonstrating its effectiveness. Similarly, in the studies conducted by the authors [44,45], neural networks achieved MAPEs ranging from 1.32% to 1.99% and 1.83% to 4.07%, respectively, for biological growth modeling. These findings highlight the potential of neural networks as an alternative approach to regression analysis for accurately modeling biological growth processes.

FL-based models have several limitations, despite their usefulness in handling uncertainty and imprecision. Foremost among them, these models can become highly complex, especially when dealing with multiple input variables and numerous linguistic rules. The more complex the system becomes, the more difficult it can become to interpret and manage it effectively. Moreover, building an accurate FL model often requires expert input to refine the linguistic variables and membership functions. However, eliciting expert knowledge can be time-consuming and subjective, leading to biases in the model. Like other AI models, FL models are also prone to overfitting if the training data does not adequately represent the entire domain. Overfitting can lead to poor generalization and inaccurate predictions. Despite these limitations, FL remains a valuable tool for certain problems, especially in the presence of uncertainty and vagueness, and when expert knowledge is readily available to build a useful model.

While this study produced noteworthy results, it is important to consider its limitations. Specifically, this study only focused on researching the use of FL for weight estimation. Future studies should explore practical approaches for the automatic weight estimation of live broiler chickens using machine vision and FL, which could have significant implications for the poultry industry.

## 5. Conclusions

Accurately estimating poultry body weight is a critical task for the poultry industry, as it directly affects farm management and profitability. To this end, researchers have explored various approaches to estimating poultry body weight, including traditional methods and advanced AI techniques. This research introduces a novel technique for the weight estimation of broiler chickens utilizing an FL approach. The developed Mamdani FL model was evaluated using different defuzzification methods to obtain the best results, with the COA method ultimately used for the defuzzification process. The FL models (FIS1, FIS2, FIS3, FIS4, FIS5, and FIS6) showed APE values (%) ranged between 0.02–5.78, 0.21–5.81, 0.12–4.84, 0.41–4.29, 0.15–3.66, and 0.37–2.47, respectively, depending on the season.

These results suggest that FL systems with Mamdani inference, which include triangular and trapezoidal membership functions of input–output variables and COA defuzzification, have potential for accurately and efficiently estimating the weight of poultry and could pave the way for further research in this field. However, there is still room for further research to develop more accurate and practical methods for automatic weight estimation of live broiler chickens using machine vision. By exploring innovative approaches, the poultry industry can continue to improve its management practices and ensure the optimal growth and productivity of poultry flocks.

## Figures and Tables

**Table 1:** The statistical parameters of the inputs and output data.

		Inputs			Output
Seasons (Dates)	Statistics	Temperature (t_i_, °C)	Relative Humidity (rh_i_, %)	Feed Consumption (fc, g)	Chicken Weight (wa, g)
S1 (3 February 2018 16 March 2018)	Minimum	20.90	57.03	189	190
	Maximum	29.95	64.40	4284	2380
	Mean	24.96	62.22	1885.50	1233.83
S2 (9 April 2018 20 May 2018)	Minimum	21.14	55.43	170	164
	Maximum	30.56	69.95	4087	2400
	Mean	25.81	63.11	1851.33	1234.33
S3 (12 June 2018 22 July 2018)	Minimum	25.96	58.43	186	182
	Maximum	30.18	70.77	4382	2420
	Mean	27.18	65.63	2021.50	1320.17
S4 (9 November 2018 19 December 2018)	Minimum	20.11	56.45	155	158
	Maximum	29.76	73.29	4239	2420
	Mean	24.52	68.66	1841.83	1186.83
S5 (9 January 2019 19 February 2019)	Minimum	20.19	58.00	169	174
	Maximum	29.55	74.00	4408	2666
	Mean	24.31	65.04	2008.83	1366.17
S6 (14 March 2019 29 April 2019)	Minimum	20.36	56.43	223	213
	Maximum	30.10	74.43	4349	2660
	Mean	24.56	67.05	2007.67	1414.83
S7 (16 July 2019 26 August 2019)	Minimum	23.06	55.70	185	196
	Maximum	30.70	73.41	4812	2640
	Mean	26.39	63.72	2209	1391.67
S8 (11 September 2019 23 October 2019)	Minimum	20.45	56.64	150	146
	Maximum	28.87	85.86	4102	2350
	Mean	25.39	70.48	1796.50	1183.50

**Table 2:** Linguistic terms for inputs and output for weeks of the rearing periods.

Fuzzy Set	Weeks	Linguistic Terms	Inputs			Output
			t_i_, °C	rh_i_, %	fc, g	wa, g
FIS1	W1	Very Low (VL)	23–27	30–50	100–160	100–160
		Low (L)	25–29	40–60	140–180	140–180
		Medium (M)	27–31	50–70	160–200	160–200
		High (H)	29–33	60–80	180–220	180–220
		Very High (VH)	31–35	70–100	200–260	200–260
		Very Low (VL)	20–24	30–50	250–400	250–400
		Low (L)	22–26	40–60	350–450	350–450
FIS2	W2	Medium (M)	24–28	50–70	400–500	400–500
		High (H)	26–30	60–80	450–550	450–550
		Very High (VH)	28–32	70–100	500–650	500–650
		Very Low (VL)	18–22	30–50	1000–1175	700–875
		Low (L)	20–24	40–60	1100–1250	800–950
FIS3	W3	Medium (M)	22–26	50–70	1175–1325	875–1025
		High (H)	24–28	60–80	1250–1400	950–1100
		Very High (VH)	26–30	70–100	1325–1500	1025–1200
		Very Low (VL)	18–21	30–50	1800–2000	1250–1375
		Low (L)	19.5–22.5	40–60	1900–2100	1300–1450
FIS4	W4	Medium (M)	21–24	50–70	2000–2200	1375–1525
		High (H)	22.5–25.5	60–80	2100–2300	1450–1600
		Very High (VH)	24–28	70–100	2200–2500	1525–1700
		Very Low (VL)	18–20.5	30–50	3000–3200	1750–1950
		Low (L)	19–22	40–60	3100–3300	1850–2050
FIS5	W5	Medium (M)	20.5–23.5	50–70	3200–3400	1950–2150
		High (H)	22–25	60–80	3300–3500	2050–2250
		Very High (VH)	23.5–27	70–100	3400–3700	2150–2400
		Very Low (VL)	16.0–18.5	30–50	4000–4300	2250–2375
		Low (L)	17.0–20.0	40–60	4150–4450	2300–2450
FIS6	W6	Medium (M)	18.5–21.5	50–70	4300–4600	2375–2525
		High (H)	20.0–23.0	60–80	4450–4750	2450–2600
		Very High (VH)	21.5–26.0	70–100	4600–4900	2525–2750

**Table 3:** Some implemented if–then rules.

MFIS	Rules	If			Then
		t_i_	rh_i_	fc	wa
FIS1	1	Low	Low	Very Low	Very Low
	2	Medium	Medium	Medium	Medium
	⁝	⁝	⁝	⁝	⁝
	10	Medium	Medium	Very High	Very High
FIS2	11	Medium	Medium	Very Low	Very Low
	12	Medium	Low	Very High	High
	⁝	⁝	⁝	⁝	⁝
	30	High	High	Low	Low
FIS3	31	Medium	Medium	Low	Low
	32	Medium	Medium	Medium	High
	⁝	⁝	⁝	⁝	⁝
	45	Medium	Low	Very Low	Very Low
FIS4	46	High	Medium	Medium	Low
	47	Medium	High	High	High
	⁝	⁝	⁝	⁝	⁝
	60	Very High	High	Very Low	Very Low
FIS5	61	Medium	High	Very High	Very High
	62	Low	Medium	Low	Low
	⁝	⁝	⁝	⁝	⁝
	77	High	Medium	Medium	Medium
FIS6	78	Low	Medium	Low	Medium
	79	Medium	Medium	Very High	Very High
	⁝	⁝	⁝	⁝	⁝
	97	High	Very High	Low	Low

**Table 4:** Summary of the measured and estimated broiler weight values.

Fuzzy Sets	Weeks	Seasons	Measured	Estimated	APE (%)
FIS1	W1	S1	190.00	189.70	0.16
		S2	164.00	170.51	3.97
		S3	182.00	189.71	4.24
		S4	158.00	163.05	3.20
		S5	174.00	170.44	2.05
		S6	213.00	212.95	0.02
		S7	196.00	189.73	3.20
		S8	146.00	154.44	5.78
FIS2	W2	S1	499.00	469.99	5.81
		S2	469.00	469.99	0.21
		S3	532.00	524.99	1.32
		S4	378.00	367.77	2.71
		S5	484.00	469.98	2.90
		S6	553.00	565.27	2.22
		S7	546.00	524.97	3.85
		S8	392.00	410.00	4.59
FIS3	W3	S1	948.00	919.91	2.96
		S2	931.00	950.00	2.04
		S3	1177.00	1130.83	3.92
		S4	869.00	870.00	0.12
		S5	1024.00	1073.52	4.84
		S6	1101.00	1069.94	2.82
		S7	1085.00	1118.43	3.08
		S8	802.00	805.12	0.39
FIS4	W4	S1	1397.00	1367.68	2.10
		S2	1466.00	1460.00	0.41
		S3	1536.00	1520.00	1.04
		S4	1400.00	1460.00	4.29
		S5	1588.00	1631.58	2.74
		S6	1689.00	1631.24	3.42
		S7	1580.00	1600.00	1.27
		S8	1379.00	1359.82	1.39
FIS5	W5	S1	1989.00	1967.76	1.07
		S2	1976.00	2001.93	1.31
		S3	2074.00	2070.80	0.15
		S4	1896.00	1965.44	3.66
		S5	2261.00	2200.03	2.70
		S6	2273.00	2200.07	3.21
		S7	2303.00	2353.53	2.19
		S8	2032.00	2071.79	1.96
FIS6	W6	S1	2380.00	2392.08	0.51
		S2	2400.00	2379.14	0.87
		S3	2420.00	2404.99	0.62
		S4	2420.00	2390.11	1.24
		S5	2666.00	2600.05	2.47
		S6	2660.00	2600.05	2.25
		S7	2640.00	2649.83	0.37
		S8	2350.00	2379.41	1.25

## Data Availability

The data presented in this study are available on request from the corresponding author.

## References

[1] Berckmans D. (2014). Precision livestock farming technologies for welfare management in intensive livestock systems. Rev. Sci. Tech..

[2] Gerland P., Raftery A.E., Ševčíková H., Li N., Gu D., Spoorenberg T., Alkema L., Fosdick B.K., Chunn J., Lalic N. (2014). World population stabilization unlikely this century. Science.

[3] Okinda C., Lu M., Liu L., Nyalala I., Muneri C., Wang J., Zhang H., Shen M. (2019). A machine vision system for early detection and prediction of sick birds: A broiler chicken model. Biosyst. Eng..

[4] USDA Livestock and Poultry: World Markets and Trade. https://apps.fas.usda.gov/psdonline/circulars/livestock_poultry.pdf.

[5] Chowdhury M.A.H., Ashrafudoulla M., Mevo S.I.U., Mizan M.F.R., Park S.H., Ha S.D. (2023). Current and future interventions for improving poultry health and poultry food safety and security: A comprehensive review. Compr. Rev. Food Sci. Food Saf..

[6] Mortensen A.K., Lisouski P., Ahrendt P. (2016). Weight prediction of broiler chickens using 3D computer vision. Comput. Electron. Agric..

[7] Nyalala I., Okinda C., Kunjie C., Korohou T., Nyalala L., Chao Q. (2021). Weight and volume estimation of poultry and products based on computer vision systems: A review. Poult. Sci..

[8] Wang L., Sun C., Li W., Ji Z., Zhang X., Wang Y., Lei P., Yang X. (2017). Establishment of broiler quality estimation model based on depth image and BP neural network. Trans. Chin. Soc. Agric. Eng..

[9] Chedad A., Aerts J.-M., Vranken E., Lippens M., Zoons J., Berckmans D. (2003). Do heavy broiler chickens visit automatic weighing systems less than lighter birds?. Br. Poult. Sci..

[10] Momoh O., Kershima D. (2010). Linear body measurements as predictors of body weight in Nigerian local chickens. ASSET Int. J. Ser. A.

[11] Narinç D., Narinç N.Ö., Aygün A. (2017). Growth curve analyses in poultry science. World Poult. Sci. J..

[12] Assan N. (2013). Bioprediction of body weight and carcass parameters from morphometric measurements in livestock and poultry. Sci. J. Rev..

[13] Zadeh L.A. (1988). Fuzzy logic. Computer.

[14] Birle S., Hussein M., Becker T. (2013). Fuzzy logic control and soft sensing applications in food and beverage processes. Food Control.

[15] Cardiel-Ortega J.J., Baeza-Serrato R. (2023). Failure mode and effect analysis with a Fuzzy Logic approach. Systems.

[16] Küçüktopcu E., Cemek B. (2021). Comparison of neuro-fuzzy and neural networks techniques for estimating ammonia concentration in poultry farms. J. Environ. Chem. Eng..

[17] Milosevic B., Ciric S., Lalic N., Milanovic V., Savic Z., Omerovic I., Doskovic V., Djordjevic S., Andjusic L. (2019). Machine learning application in growth and health prediction of broiler chickens. World Poult. Sci. J..

[18] Okinda C., Nyalala I., Korohou T., Okinda C., Wang J., Achieng T., Wamalwa P., Mang T., Shen M. (2020). A review on computer vision systems in monitoring of poultry: A welfare perspective. Artif. Intell. Agric..

[19] Wu D., Cui D., Zhou M., Ying Y. (2022). Information perception in modern poultry farming: A review. Comput. Electron. Agric..

[20] Bao J., Xie Q. (2022). Artificial intelligence in animal farming: A systematic literature review. J. Clean. Prod..

[21] Ojo R.O., Ajayi A.O., Owolabi H.A., Oyedele L.O., Akanbi L.A. (2022). Internet of Things and Machine Learning techniques in poultry health and welfare management: A systematic literature review. Comput. Electron. Agric..

[22] Küçüktopcu E., Cemek B. (2019). Evaluating the influence of turbulence models used in computational fluid dynamics for the prediction of airflows inside poultry houses. Biosyst. Eng..

[23] Chen G., Pham T.T., Boustany N. (2001). Introduction to fuzzy sets, fuzzy logic, and fuzzy control systems. Appl. Mech. Rev..

[24] Zadeh L.A. (1975). The concept of a linguistic variable and its application to approximate reasoning—I. Inf. Sci..

[25] Alojaiman B. (2023). A Multi-Criteria Decision-Making Process for the Selection of an Efficient and Reliable IoT Application. Processes.

[26] Moharrery A., Kargar A. (2007). Artificial Neural Network for prediction of plasma hormones, liver enzymes and performance in broilers. J. Anim. Feed Sci..

[27] Ahmad H. (2009). Poultry growth modeling using neural networks and simulated data. J. Appl. Poult. Res..

[28] Demmers T.G., Cao Y., Gauss S., Lowe J.C., Parsons D.J., Wathes C.M. (2010). Neural predictive control of broiler chicken growth. IFAC Proc. Vol..

[29] Ahmadi H., Golian A. (2010). Growth analysis of chickens fed diets varying in the percentage of metabolizable energy provided by protein, fat, and carbohydrate through artificial neural network. Poult. Sci..

[30] Ghazanfari S. (2014). Application of Linear Regression and Artificial NeuralNetwork for Broiler Chicken Growth Performance Prediction. Iran J. Appl. Anim. Sci..

[31] Johansen S.V., Bendtsen J.D., Martin R., Mogensen J. (2017). Data driven broiler weight forecasting using dynamic neural network models. IFAC-Pap..

[32] Pedrozo Abreu L.H., Yanagi Junior T., Bahuti M., Lima R.R.d., Lourençoni D., Fassani É.J. (2019). Performance of broilers submitted to different intensities and duration of thermal stress. Dyna.

[33] Zadeh L.A. (1965). Fuzzy sets. Inf. Control.

[34] Rahimi H., Nazemizadeh M. (2014). Dynamic analysis and intelligent control techniques for flexible manipulators: A review. Adv. Robot..

[35] Castillo O., Melin P. (2014). A review on interval type-2 fuzzy logic applications in intelligent control. Inf. Sci..

[36] Mamdani E.H., Assilian S. (1975). An experiment in linguistic synthesis with a fuzzy logic controller. Int. J. Man-Mach. Stud..

[37] Chen C.-M. (2009). A fuzzy-based decision-support model for rebuy procurement. Int. J. Prod. Econ..

[38] Bottani E., Gentilotti M.C., Rinaldi M. (2017). A fuzzy logic-based tool for the assessment of corporate sustainability: A case study in the food machinery industry. Sustainability.

[39] El Jaouhari A., El Bhilat E.M., Arif J. (2023). Scrutinizing IoT applicability in green warehouse inventory management system based on Mamdani fuzzy inference system: A case study of an automotive semiconductors industrial firm. J. Ind. Prod. Eng..

[40] Klir G.J., St. Clair U., Yuan B. (1997). Fuzzy Set Theory: Foundations and Applications.

[41] Franzo G., Legnardi M., Faustini G., Tucciarone C.M., Cecchinato M. (2023). When Everything Becomes Bigger: Big Data for Big Poultry Production. Animals.

[42] Leishman E., You J., Ferreira N., Adams S., Tulpan D., Zuidhof M., Gous R., Jacobs M., Ellis J. (2023). When worlds collide–Poultry modelling in the ‘Big Data’era. Animal.

[43] Amraei S., Abdanan Mehdizadeh S., Salari S. (2017). Broiler weight estimation based on machine vision and artificial neural network. Br. Poult. Sci..

[44] Küçüktopcu E., Cemek B. (2021). Comparative analysis of artificial intelligence and nonlinear models for broiler growth curve. Int. J. Agric. Wild. Sci..

[45] Roush W., Dozier W., Branton S. (2006). Comparison of Gompertz and neural network models of broiler growth. Poult. Sci..

